# Sources of financing: Which ones are more effective in innovation–growth linkage?

**DOI:** 10.1016/j.ecosys.2023.101177

**Published:** 2024-06

**Authors:** Anabela M. Santos, Michele Cincera, Giovanni Cerulli

**Affiliations:** a European Commission, Joint Research Centre, Edificio EXPO, Calle Inca Garcilaso 3, 41092 Seville, Spain; b Université libre de Bruxelles, Solvay Brussels School of Economics and Management, Times², Brussels, Belgium; c Research Institute on Sustainable Economic Growth, National Research Council of Italy, Rome, Italy

**Keywords:** Financing, Innovation, Growth, Europe

## Abstract

The study assesses the impact of eight sources of financing (internal funds, bank loans, credit lines, trade credit, equity, grants, leasing and factoring) on innovation and firm growth. It provides evidence that not all external financing sources have the same impact on innovation and growth. Output additionality on turnover growth seems higher for equity financing. In contrast, employment growth appears to be more associated with financing sources linked to increased fixed assets or the solving of liquidity problems. The number of financing instruments used together also seems to matter, revealing the existence of complementarities.

## Highlights

● Access to finance has a positive impact on innovation and, consequently, on growth.
● Not all sources of external finance have the same impact on innovation and growth.
● Turnover growth is higher for equity finance than for other sources.
● Grants have the greatest impact on employment growth.
● Complementarities between the use of different sources of finance were found.

## 1 Introduction

The financing–innovation–growth nexus is a multistage process. More specifically, access to finance has a first-step leverage impact on innovation and a second-step impact linking additional innovation to growth. According to Levine (2005), innovation can foster growth only in the presence of a financial system, and this is also the central pillar of Schumpeterian theory (Schumpeter, 1934). Schumpeter (1934) was the first scholar to support a positive relationship between innovation and growth sustained by financial services and intermediaries. According to Schumpeterian theory, through entrepreneurship and credit system intermediation, innovation can sustain economic growth, as successful innovation gives firms a competitive advantage in the market vis-à-vis competitors. The financing–innovation–growth linkage was also supported by King and Levine (1993). These authors show that a better financial system improves the probability of successful innovation, thus accelerating economic growth.

Furthermore, capital market imperfections, such as asymmetric information between lenders and borrowers and other agency costs or moral hazard problems, influence companies’ capital investment decisions and introduce possible financing constraints (i.e. credit rationing by lenders). These constraints may be even more severe in the case of intangible investments, such as research and development (R&D), since these activities are riskier by nature and typically provide less collateral to lenders than capital goods do (Cincera and Ravet, 2010).

For these reasons, innovative firms face more severe constraints and barriers to accessing external financing (Lee et al., 2015, Cincera et al., 2016, Santos and Cincera, 2022), for their projects have higher levels of uncertainty and risk and there are fewer assets to use as collateral (Hall and Lerner, 2010). Therefore, innovative firms need to find alternative channels when banks refuse to provide loans and when internal sources of financing are insufficient. Some available options, depending on the type of project to be financed, are (i) finding private equity (PE) funds, such as business angels and venture capitalists; (ii) applying for a government grant or subsidy; (iii) asking for trade credit from suppliers; (iv) obtaining asset-based financing, such as leasing; (v) selling the firm’s invoices to a factoring company to obtain liquidity more quickly; or (vi) trying to collect funds on the internet using crowdfunding. This diversification and choice of financing sources have been shown to be intrinsically related to whether a firm faces financing constraints (Chit and Rizov, 2023). Although financing constraints are not the main focus of this paper, it is important to highlight that the choice of financing instruments depends on firm and project characteristics, which influence the severity of the financing constraints (see, for example, Santos and Cincera, 2022).

The paper aims to explore the impact of eight sources of financing (internal funds, bank loans, credit lines, trade credit, equity, grants, leasing and factoring) on innovation and then on firm growth. It aims to provide empirical evidence of which source of financing is most effective at enhancing innovation and growth. For this purpose, we use the anonymous Survey on the Access to Finance of Enterprises (SAFE), and we combine the results of two waves to follow the evolution of the 3 786 small and medium-sized enterprises (SMEs) in the EU between 2014 and 2015. The methodological approach is based on a three-step procedure. The first two steps are based on propensity score matching (PSM) and estimate the impact^1^ of financing on innovation. The third step takes the form of a regression estimation, considering the impact of innovation financing as an explanatory variable of firm growth.

The contribution and originality of the paper take several forms. First, since the seminal contributions of Schumpeter (1934) and King and Levine (1993), few studies have simultaneously assessed the relationships among these three components (finance, innovation and growth). Most existing studies have focused more on evaluating the impact of innovation on firm growth (e.g., Coad et al., 2016), financing on innovation (e.g., Yano and Shiraishi, 2020) or financing on growth (e.g., Bangake and Eggoh, 2011; Cortina et al., 2018; Banai et al., 2020; Busu et al., 2021; Silva et al., 2021). Second, the paper intends to assess the impact of eight sources of financing at the same time, making their impacts comparable. Usually, the scientific literature focuses more on assessing the impact of only one source of financing (e.g. public support, venture capital (VC) or bank loans) on innovation or firm growth. However, there are few comparisons of the impacts of different sources of finance^2^. Given the lack of empirical evidence, a better understanding of which sources of external funding are the most effective for spurring innovation and, subsequently, economic growth should be of primary interest for innovative firms in their strategies for financing their innovative activities. Third, we used a database that, as far as we know, has not previously been used for this purpose.

The paper is structured in five sections. After the introduction, Section 2 summarises the main existing findings about the financing–innovation–growth linkage in the scientific literature. Section 3 presents the framework, methodology and data used. Section 4 describes the results obtained. Section 5 provides the conclusions and some policy recommendations.

## 2 Review of the literature

### 2.1 Innovation and sources of financing

Investments in intangibles, such as R&D, are riskier by nature than ordinary investments, and R&D managers often have better information regarding the likelihood of the success of their R&D projects than outside investors or lenders do (Hall**,** 2009)^3^. Banks are central players in the financial market, and they lend money to firms under specific conditions, for either growth or day-to-day business operations (Silva et al., 2021). Nevertheless, access to bank finance is subject to a selection process, and the primary determinant for the lender’s decision is the firm’s and investment project’s levels of risk (Savignac, 2007). Because of uncertainty and risk, traditional financial markets and financial institutions are more reluctant to invest in R&D projects than in conventional business projects (Mazzucato, 2013). This situation is often due to asymmetric information: when one of the parties has better information about the project than the other. In addition, asymmetric information also increases the cost of external financing (Hall**,** 2009).

Owing to the existence of market failures, innovative firms are typically more financially constrained (Santos and Cincera, 2022) and, given the imperfections in markets for equity and debt, not all firms access external capital markets to finance their R&D investments with the same ease and conditions. Furthermore, the choice of financing instruments depends on several factors. First, between internal and external financing, small innovative firms tend to prefer internal financing (Magri**,** 2009); however, only financially unconstrained firms can act on this preference (Chit and Rizov, 2023). Second, when firms do not have internal capacity, debt is revealed to be preferable to equity, as the former involves less loss of control rights (Casson et al., 2008). However, equity investors, such as business angels or venture capitalists, are more likely to finance riskier projects than banks are (Cincera and Santos, 2015).

In addition to the above sources of finance, there are others that innovative firms can use as alternative or complementary sources of finance (Table 1). For instance, trade credit can be an alternative source of short-term external finance for credit-constrained firms, particularly innovative SMEs (Bönte and Nielen, 2011). Firms with high intangible assets are more likely to use trade credit (Tsuruta, 2008). Demand for and supply of trade credit are also higher for innovative firms (Nielen, 2016). Firms that are credit rationed usually ask for trade credit as a substitute for bank debt, leading to higher demand for these financing instruments (Nilsen, 2002). In addition, since suppliers have closer relationships with their customers and, consequently, more information about their business, the level of asymmetric information can be reduced (Petersen and Rajan, 1997), leading to greater availability of trade credit for innovative firms (Nielen, 2016).

**Table 1:** Main financing sources by type of expenditure.

Instrument	Description	Main purpose		
		R & D activity	Fixed assets	Working capital
Internal funds	Cash or cash equivalent, resulting, for instance, from savings, retained earnings or sale of assets	**✓**	**✓**	**✓**
Bank loans	Long-term debt in exchange for its reimbursement and a financing cost (interest rate)		**✓**	
Credit lines	Short-term loans that can be used fully or partially, at discretion, up to a pre-arranged limit	Could be if included in day-to-day expenditure (e.g. to pay wages)		**✓**
Private equity	Equity financing obtained in exchange for a share of firm ownership	**✓**	**✓**	**✓**
Grants or subsidy	Non-reimbursable funds, low-interest loans or interest-free loans provided by the government	**✓**	**✓**	
Trade credit	To pay suppliers of goods, services or equipment at a later, agreed date	Only if done outside the firm	**✓**	**✓**
Leasing	To make regular payments in exchange for the use of a fixed asset, without its immediate ownership		**✓**	
Factoring	To sell firm invoices (debt from customers) to a factoring company in exchange for immediate cash, but at a lower amount than their value	Generate cash flow for any purpose		

NB: Description of financial instrument is based on SAFE questionnaire description.

*Source:* Created by the authors.

Leasing can also be a substitute for traditional bank debt for new firms that lack collateral, since asset ownership remains with the lessor^4^ and is not transferred to the customer (lessee), who only has the right to use it during a specified period in exchange for regular payments (OECD**,** 2015b). Furthermore, firms choose to use leasing because this asset-based finance lets them manage their working capital better by paying for the equipment over the life of the asset and not all at once at the beginning^5^ (Oxford Economics, 2015). Similarly, firms can also use factoring as a financing source for working capital and as an instrument to improve cash flow (Soufani, 2001). Factoring is revealed to be particularly useful for high-risk firms and for firms with high investments in intangible assets that cannot use other types of financing instruments (OECD**,** 2015b).

However, it is important to stress that the use and combination of different sources of finance also depend on the level of the firm’s financing constraints. According to Chit and Rizov (2023), SMEs with moderate financing constraints use more diversified sources of finance, while SMEs with severe financing constraints can only afford less-diversified sources of finance.

### 2.2 Financing, innovation and growth

As regards the findings in the literature dealing with the first stage of the financing–innovation–growth linkage, while the theory argues that access to finance is a driver of innovation (Schumpeter, 1934, King and Levine, 1993, Levine, 2005), the results of empirical analyses of the impact of different sources of finance on R&D and innovation diverge.

For instance, government subsidies can stimulate and trigger innovation measured as private R&D effort (Busom, 2000, González et al., 2005, Cerulli and Potì, 2012, Huergo et al., 2016), the number of patent applications (Bronzini and Piselli, 2016), intangible assets (Yano and Shiraishi, 2020) and the likelihood of introducing a new product or process (Batterink et al., 2006, Foreman-Peck, 2013). Nevertheless, the literature also reveals that public support can have an adverse effect, especially when it substitutes or crowds out^6^ private R&D expenditure (Dai and Cheng, 2015, Marino et al., 2016). Furthermore, it could also have no impact^7^ on the development of new products (Hashi and Stojčić, 2013, López-Fernández et al., 2011, Benavente et al., 2012), and on the number of patents registered (Benavente et al., 2012).

Studies on PE financing’s impact on firm innovation performance also report divergences. Some authors (e.g., Kortum and Lerner, 2000; Popov and Roosenboom, 2009; Amess et al., 2016) found a positive impact of PE financing on innovation, whereas others (e.g., Engel and Keilbach, 2007; Capizzi et al., 2011; Guo and Jiang, 2013) show some evidence that PE does not improve innovation.

Some explanations highlighted are that VC seems to focus on the commercialisation of existing innovations and firm growth (Engel and Keilbach, 2007). Furthermore, according to Peneder (2010), the higher innovation performance of VC-financed firms is the result of the VC selection process and not a direct causal effect of VC financing on innovation. According to the author, this means that venture capitalists tends to finance and select firms that already have an above-average level of innovation rather than make firms more innovative. Certainly, if the main goal of VC is to maximise financial return (Metrick and Yasuda, 2010), this source of funding can be expected to focus more on the profitability of investments that have already been made and have market potential rather than on more uncertain project investments.

Regarding the impact of debt financing instruments (such as bank loans, credit lines, trade credit, leasing and factoring) on firms’ innovation performance, the (less-extensive) literature seems to converge to similar conclusions. Bank debt has a positive impact on R&D activity (e.g., Benfratello et al., 2008; Hikmi and Parnaudeau, 2008), and non-bank financing is a complementary instrument for alleviating financing constraints (Bönte and Nielen, 2011, Kraemer-Eis and Lang, 2012, OECD, 2015b).

As regards findings in the literature dealing with the second step of the financing–innovation–growth linkage, despite theory pointing to a positive relationship between innovation and firm growth or performance (Schumpeter, 1934, King and Levine, 1993, Levine, 2005), some studies found a negative or non-significant relationship (Mairesse and Mohnen, 2010, Mohnen and Hall, 2013). However, despite some divergences in conclusions about the direction and significance of the impacts, most studies conclude that innovation is positively associated with firms’ growth or performance but is highly context dependent (Rosenbusch et al., 2011). According to Mohnen and Hall (2013), the complementarity between different types of innovation could justify why, when estimated together, some are non-significant or produce a negative coefficient. Indeed, the simultaneity of different types of innovation makes it difficult to isolate the individual effect of each type (Mohnen and Hall, 2013), and a way to mitigate this complementarity problem could be to combine the dichotomous measures of innovation type with the innovation strategies.

## 3 Data and methodology

**Fig. 1:**
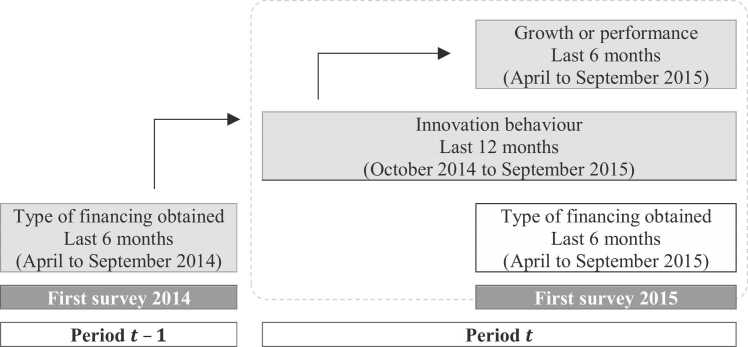
Timeline of the database.

Source: Created by authors based on SAFE template.

To perform our analysis, data was taken from the anonymous^8^ SAFE, conducted by the European Central Bank and the European Commission since 2009. Data from the first surveys of 2014 and 2015^9^ was used to build a panel, leading to an assessment of firms’ growth patterns^10^. These two surveys cover the period from April 2014 to September 2015 (Fig. 1). After selecting only firms with valid answers to all the dimensions included in the analysis, our sample contains 3 786 SMEs in the EU-28.

The central hypothesis in the timeline used in the study is that financing obtained (or used) in a period (*t – 1*) could influence firms’ capacity to introduce an innovation in the market (product or service) or their organisation (process or marketing) in the next period (*t*)^11^. Information about financing covers 6 months (April to September 2014), whereas information about innovation strategy refers to the subsequent 12 months (October 2014 to September 2015). A posteriori, as the result of innovation behaviour, firms can grow in terms of turnover or employment level (April to September 2015). The choice of sales and employment as variables to measure growth is due to the information available in the SAFE database regarding the firms’ growth indicators.

As the information on the innovation strategy in the SAFE database refers to the last 12 months, whereas that on financing refers to the last 6 months, this means that when using only one wave of the SAFE database we are faced with a situation where innovation can take place before new financing is issued. Therefore, to observe the impact of financing on innovation, we need to combine two waves of SAFE, as shown in Fig. 1, to ensure that access to new financing sources happens before innovation. Growth is expected to happen just after the innovation or even simultaneously with it. An extended period is not necessary before recording at least turnover growth, for example, due to introducing a new product in the market.

To assess the impact of financing on innovation behaviour and firm growth, we consider a conceptual framework similar to that used by Cerulli and Potì (2012) based on a three-step approach. The first two steps are based on the PSM and estimate the effect of financing on innovation, considering the endogenous nature of using a specific source of finance. This endogeneity occurs because access to external funding is subject to a selection process (Busom, 2000, Peneder, 2010). The third step takes the form of a regression estimation, considering the effect of innovation financing as an explanatory variable of firm growth.

Propensity score matching is used to estimate the impact of a specific policy through its average treatment effect (ATE). The ATE (see Eq. (1)) corresponds to the difference between the average of the outcome (or target variable) when the firm is treated ($y_{1}$) and the average of the outcome (or target variable) when the same firm is untreated ($y_{0}$). The treatment could refer to a situation where a firm receives a grant or obtains another source of financing. In this study, the outcome (or target variable) is the decision of a firm to innovate (i.e. the firm’s innovation behaviour). However, the impact assessment literature has generally focused on estimating the average treatment effect on the treated (ATET), which corresponds to the difference between the outcome with and without treatment for those who were treated (Caliendo and Kopeining, 2008), as expressed in Eq. (2), with *w = 1* corresponding to being treated.

(1) $$\mathrm{ATE}=E\left(y_{1}\right)-E(y_{0})$$

(2) $$\mathrm{ATET}=E\left(y_{1}|,w=1\right)-E(y_{0}|w=1)$$

One main issue in estimating the ATET is that we can only observe $y_{1}$; $y_{0}$ is unknown, as we cannot simultaneously observe the treated and untreated status (*w*) of the same firm. To estimate the effect of the treatment, we can use a matching procedure. For each observation, ‘matching estimators impute the missing outcome by finding other individuals in the data whose covariates are similar but who were exposed to the other treatment’ (Abadie et al., 2004, p. 292). Similarity among individuals is measured using units’ distances in terms of their conditional treatment probabilities, also called the ‘propensity score’ (see Eq. (3)), usually estimated using a binary choice model, such as logit or probit.

(3) $$p\left(x\right)=\mathrm{prob}(w=1|x)$$

The two main identification assumptions when using matching are the overlapping assumption and the conditional mean independence (CMI) assumption. The overlapping assumption, also known as the ‘common support condition’, assumes that 0 < $p\left(x\right)\;$< 1. If this assumption does not hold, there might be units with a specific characteristic x that either always receive treatment or never receive treatment (Cerulli, 2015, p. 71), making it impossible to identify the ATET because units have no proper comparison individual in the opposite treatment group. One way to check the overlap assumption is through visual analysis of the propensity score density distribution in both groups, treated and untreated: ideally, the two distributions would not to be too polarised, thus showing sufficient frequencies in the common region of the propensity score values. The CMI assumption (Eq. (4)) assumes that the mean of $y_{0}$ given x does not depend on the treatment variation (w), which implies that this mean is the same for any value of w (Cerulli, 2015, p. 70). The CMI is not directly testable, as it depends on the treatment selection process (Khandker et al., 2010, p. 56). To ensure this assumption holds, Caliendo and Kopeining (2008, p. 38) suggest that covariates should be fixed over time or measured before participation. Under the CMI assumption, we have the following.

(4) $$E\left(y_{0}\right|w=1,x)=E(y_{0}|w=0,x)$$

This entails that – conditional on the covariates – the observable expectation in the right-hand side of Eq. (4) can identify the unobservable expectation on the left-hand side. Moreover, in the third-step analysis, we will measure the idiosyncratic innovation policy effect by an estimation of *ATET*(*x*_*i*_) (Eq. (5)).

(5) $$\mathrm{ATET}\left(x_{i}\right)=E\left(y_{1i}-y_{0i}|,w_{i}=1,x_{i}\right)={\;\mathrm{ATE}}_{\left(w_{i}=1\right)}(x_{i})$$

As the quantity to be estimated in Eq. (5) is specific to each firm, we will indicate it using simply *ATET*_*i*_.

In the present study, the outcome variable corresponds to firm innovation behaviour in period t, measured by the number of innovation types launched in the market or the firm’s organisation. This accounts for the complementarity among different types of innovation, as suggested by Mohnen and Hall (2013). Firms can jointly or separately introduce four types of innovation: (i) product or service, (ii) production process or method, (iii) organisation of management or (iv) marketing (a new way of selling goods or services). In addition, the dichotomous variable (yes or no) used to measure innovation is also included in the analysis as an outcome of a robustness test.

The treatment variable coincides with the status of receiving, obtaining or using the following sources of financing, jointly or separately, in period *t – 1*: (i) internal funds; (ii) bank loans; (iii) credit lines, bank overdrafts, or credit card overdrafts; (iv) trade credit; (v) equity capital; (vi) grants or subsidised bank loans; (vii) leasing or hire purchase; and (viii) factoring. On average, firms included in the control group did not obtain new financing in *t – 1*, except for internal funds and leasing.

The covariates to include in the propensity score model (Eq. (3)) are those usually listed in the literature and common to all sources of financing: (i) firm size, (ii) firm age, (iii) the firm’s primary ownership, (iv) autonomy or belonging to a group, (v) export intensity, (vi) firm performance, (vii) activity sector and (viii) country. For more details of the variable description, see Table A1 in the supplementary material. Firm size, export intensity and firm performance refer to a period before the treatment. Since the answers provided in the survey about financing instruments refer to the period between April and September 2014, firm size (measured by turnover) and export intensity concern 2013. Firm performance reports average growth performance over the past 3 years (2011–2013). Firm age, the firm’s primary ownership, autonomy or belonging to a group, activity sector and country are characteristics of firms not affected by the treatment^12^.

After estimating the ATET and testing the balancing quality^13^, this parameter – conditional on all the covariates considered – can be used as an explanatory variable for firm growth^14^. Therefore, the third and final step consists of assessing the effect of innovation additionality (ATET) on firm growth using regression analysis. In this last step, the outcome of interest (the dependent variable) is a binary variable, which assumes the value of 1 if firms have positive growth, and 0 otherwise. The binary choice model (Eq. (6)) explains the probability of having essentially $y_{i}=1$, taking into account the set of explanatory variables $x_{i}$.

(6) $$P\left\{y_{i}=1|x_{i}\right\}=G\left(x_{i},\beta \right)$$

The modelling of binary data is usually done using logit, probit or complementary log-log (Clog-log) models, where the differences lie in the estimation of function $G\left(.\right)$, also called the link function. In the present study, a Clog-log regression model was used to estimate the probability of firm growth as expressed in Eq. (7). Of the three possibilities of a link function for binary choice models reported in Table 3, we selected the Clog-log model because it fits data well in comparison with the other models, namely in terms of pseudolikelihood and the test of joint significance (results available upon request). Only firms subject to the treatment are included in the regression, as we are only interested in estimating the effect of innovation financing on growth. Thus, we consider in the regression model only firms that received or used financing.

(7) $$\mathrm{Pr}\left\{{\mathrm{growth}}_{i,t}=1|x_{i}\right\}=G\left[\beta_{0}+\beta_{1}{\mathrm{ATE}}_{i,t}+\beta_{2}{\mathrm{growth}}_{i,t-1}+\beta {\mathrm{firms}\;\mathrm{characteristics}}_{i,t}\right]$$

Firms’ growth is measured as increase in turnover and number of employees over the last 6 months. In addition to the ATE of financing on innovation, the regression also included growth status reported in a similar period 1 year before, and firm characteristics, such as firm size and age, activity sector and the region^15^ where firms are located.

## 4 Results and discussion

### 4.1 Data description

The sample is composed of 3 786 firms located in the EU-28 (for the geographical distribution of the sample, see Table B1 in the supplementary material) and operating in the industry (27.6%), construction (11.5%), trade (28.5%), and services (32.4%) sectors. Micro-sized enterprises represent around 54%, small 27.6%, and medium-sized 18.7% of the total. About 79.6% of the surveyed firms have been operating in the market for more than ten years. Young firms (under five years old) represent 7.4% of the sample, while mature firms (between 5 and 10 years old) are about 13%.

Approximately 403 firms belong to a group (10.6%). Around 83.7% are owned mainly by one person, family, or group of entrepreneurs. Firms listed in the stock market account for only a tiny proportion of the sample (2.2% of the total).

Regarding firms’ past performance, 41% said their turnover growth was less than 20% per year between 2011 and 2013. About 15% revealed an average sales growth of over 20% per year in the past three years. The rest of the sample (44%) registered no growth or even a decrease during this period. The export intensity in the year before treatment is on average 18.1%, with some firms exporting all their goods and services and others having no international behaviour.

Firms introducing an innovation to the market or in their managerial organisation represent about 60% of the sample. Some firms have implemented four innovation types (product, service, process and marketing). In the year following the use or receipt of financing, about 42% of firms increased their turnover and around 25% increased their number of employees. We also observe strong heterogeneity in terms of innovation behaviour and economic performance between countries (Table B3 in supplementary material). The percentage of SMEs introducing an innovation to the market or in their managerial organisation varies from 34.6% (Hungary) to 83.3% (Cyprus). The percentages of firms increasing their turnover and employment level range from 15.4% (Estonia) to 61.7% (Ireland) and from 15.4% (Estonia) to 52.4% (Malta), respectively.

Concerning the use of financing instruments (Table 2), about 27.7% of the sample (1 049 firms) indicated they had not used or obtained any financial instrument. The remaining 2 737 firms (72.3%) used at least one source of financing on its own or with others. The financing instruments most frequently used are leasing (30%), internal funds (17.5%) and bank debt (long-term loans, 15.7%; credit lines, 15.7%). Equity financing (3.4%) and factoring (7.7%) are the least used or obtained. The likelihood of using at least one of the different financing instruments is also greatly different across the EU-28 (see Table B2 in supplementary material). For instance, the percentage of firms using internal financing is the highest in Estonia, while percentages are highest for grants in Italy, equity and factoring in Slovakia, leasing in Germany, bank loans in France, trade credit in Spain and credit lines in Luxembourg.

**Table 2:** Financing instruments by innovation behaviour.

Variable	Whole sample		Innovative firm		Non-innovative firm		Difference in means	Relative difference^a^
	Mean	Std dev.	Mean	Std dev.	Mean	Std dev.		
No use of / request for financing	0.277	0.448	0.252	0.434	0.315	0.465	– 0.063 * **	– 20%
All financing	0.723	0.448	0.748	0.434	0.685	0.465	0.063 * **	9%
Number of instruments used	1.640	1.506	1.762	1.542	1.459	1.433	0.302 * **	21%
Internal funds	0.175	0.380	0.194	0.395	0.147	0.354	0.047 * **	32%
External financing	0.686	0.464	0.711	0.453	0.650	0.477	0.061 * **	9%
Bank loans	0.157	0.364	0.167	0.373	0.144	0.351	0.023 *	16%
Credit lines	0.157	0.364	0.168	0.374	0.142	0.349	0.026 * *	18%
Trade credit	0.105	0.307	0.116	0.321	0.088	0.284	0.028 * **	32%
Equity	0.034	0.180	0.042	0.201	0.021	0.143	0.021 * **	101%
Grants	0.113	0.316	0.129	0.335	0.089	0.285	0.040 * **	45%
Leasing	0.302	0.459	0.316	0.465	0.281	0.450	0.035 * *	13%
Factoring	0.077	0.267	0.090	0.287	0.058	0.233	0.033 * **	57%

NB: Std dev., standard deviation. Number of observations: whole sample = 3 786; innovative firms = 2 258; non-innovative firms = 1 528. All variables are dummy variables with minimums equal to 0 and maximums equal to 1, except for the number of financing types used, where the values are between 0 and 9. Number of financing instruments includes the eight sources of financing under analysis, plus a ninth category corresponding to all other sources of financing. Significance levels: * ** *p* < 0.01; * * *p* < 0.05; * *p* < 0.1.

*Source:* Authors’ calculations.

a Relative difference = ((mean innovative firms / mean non-innovative firms) – 1)

On average, innovative firms use different sources of financing 9% more than non-innovative firms, and they are also more likely to use financing (74.8%) than their non-innovative counterparts (68.5%). More significant differences between groups can be observed in equity financing, factoring and grants: innovative firms show a higher propensity to use or obtain them (between 45% and 101%) than non-innovative firms. Concerning the other types of financing sources, innovative firms also show a tendency to use them, but with a lower relative difference (13% to 32%).

On average, firms used 1.6 financing instruments (Table 2), and only 496 firms used just one of the eight types of financing instruments under analysis here (Table 3). A credit line is the source of financing used on its own the least (1.7%) and is used primarily with two (30.2%) or three (25.8%) other instruments. On the other hand, internal funds, leasing and equity are more frequently used on their own (20.8%, 15.9% and 13.4%, respectively) or combined with only one other instrument (22.8%, 32.0%, and 29.9%, respectively). Other financing instruments are more often combined with two or three other instruments.

**Table 3:** Financing instruments: used alone or in combination with others.

Instruments	Number of firms	On its own		With one other		With two others		With three others		With more than three others	
		n	%	n	%	n	%	n	%	n	%
Internal funds	663	138	20.8	151	22.8	151	22.8	110	16.6	113	17.0
Bank loans	596	50	8.4	107	18.0	151	25.3	151	25.3	137	23.0
Credit lines	596	10	1.7	115	19.3	180	30.2	154	25.8	137	23.0
Trade credit	398	41	10.3	62	15.6	94	23.6	97	24.4	104	26.1
Equity	127	17	13.4	38	29.9	27	21.3	26	20.5	19	15.0
Grants	427	36	8.4	89	20.8	99	23.2	99	23.2	104	24.4
Leasing	1 143	182	15.9	366	32.0	276	24.1	181	15.8	138	12.1
Factoring	292	22	7.5	58	19.9	68	23.3	69	23.6	75	25.7

NB: Overall, 496 firms use only one type of financing instrument. The combination of the eight financing instruments with others also included the category ‘all other sources of financing’.

*Source:* Authors’ calculations.

Concerning the combination of only two financing instruments, Table 4 shows that firms using internal funds seem to combine this mainly with leasing (45%). In contrast, grants are more associated with bank loans (45%). Trade credit and equity are more often used in conjunction with internal funds (32% and 41%, respectively), whereas factoring is more associated with leasing (56%).

**Table 4:** Number of firms using a combination of two financing instruments (only and not exclusively).

Additional source of financing	Primary source of financing															
	Internal funds		Bank loans		Trade credit		Credit lines		Equity		Grants		Leasing		Factoring	
	*n*	% TT	*n*	% TT	*n*	% TT	*n*	% TT	*n*	% TT	*n*	% TT	*n*	% TT	*n*	% TT
**Only one extra**																
Internal funds			14	20.6	12	31.6	1	16.7	9	40.9	8	16.3	38	37.3	2	7.4
Bank loans	14	16.7			9	23.7	2	33.3	0	0.0	22	44.9	20	19.6	1	3.7
Trade credit	12	14.3	9	13.2			0	0.0	3	13.6	1	2.0	8	7.8	5	18.5
Credit lines	1	1.2	2	2.9	0	0.0			1	4.5	0	0.0	2	2.0	0	0.0
Equity	9	10.7	0	0.0	3	7.9	1	16.7			2	4.1	5	4.9	2	7.4
Grants	8	9.5	22	32.4	1	2.6	0	0.0	2	9.1			14	13.7	2	7.4
Leasing	38	45.2	20	29.4	8	21.1	2	33.3	5	22.7	14	28.6			15	55.6
Factoring	2	2.4	1	1.5	5	13.2	0	0.0	2	9.1	2	4.1	15	14.7		
***Subtotal***	***84***	***100***	***68***	***100***	***38***	***100***	***6***	***100***	***22***	***100***	***49***	***100***	***102***	***100***	***27***	***100***
**One extra (but not exclusively)**																
Internal funds			152	14.6	128	17.7	140	14.4	35	19.2	106	14.2	265	21.0	73	13.8
Bank loans	152	16.9			124	17.2	234	24.1	21	11.5	201	26.9	229	18.1	82	15.5
Trade credit	128	14.2	124	11.9			143	14.7	20	11.0	80	10.7	166	13.1	61	11.6
Credit lines	140	15.6	234	22.4	143	19.8			22	12.1	124	16.6	228	18.1	80	15.2
Equity	35	3.9	21	2.0	20	2.8	22	2.3			20	2.7	49	3.9	15	2.8
Grants	106	11.8	201	19.3	80	11.1	124	12.8	20	11.0			162	12.8	53	10.0
Leasing	265	29.5	229	22.0	166	23.0	228	23.5	49	26.9	162	21.7			164	31.1
Factoring	73	8.1	82	7.9	61	8.4	80	8.2	15	8.2	53	7.1	164	13.0		
***Subtotal***	***899***	***100.0***	***1 043***	***100***	***722***	***100***	***971***	***100***	***182***	***100***	***746***	***100***	***1 263***	***100***	***528***	***100***

NB: The table reports the number of firms that use a primary source of financing (column titles) in combination with other sources of financing (row titles). Only one extra means that firms used only two of the eight types of financing instruments assessed in the study. One extra but not exclusively refers to firms that used at least two types of financing instruments. The percentage of the total (% TT) refers to the percentage estimated using as the total the value reported in the row subtotal.

*Source:* Authors’ calculations.

### 4.2 Impact of financing on innovation

Table 5 reports the results of the PSM, which corresponds to the effect of financing (measured through different sources) on firm innovation behaviour, compared with a situation where firms did not obtain or use new financing in the previous year. Using the kernel density plots’ distribution of the propensity score (Figure D1 in the supplementary material), the balancing quality test shows that covariates are balanced, as the distribution of the propensity score is very similar in both groups (financially supported firms and non-supported ones) after matching. From the graphs in Figure D1 (see the supplementary material), it is also possible to conclude that the overlapping assumption holds, as the propensity scores assume low frequencies close to 0 or 1.

**Table 5:** Effect of sources of financing in period t – 1 on innovation behaviour in period t.

Source of financing	Number of firms		Being an innovative firm	Number of innovations introduced
	Treated	Control	Yes/no	0–4
Internal funds	663	1 049	0.049 (0.034)	0.057 (0.087)
External sources	2 599	1 049	0.053 (0.024)* *	0.177 (0.055)* **
Bank loans	596	1 049	0.032 (0.039)	0.167 (0.101)*
Credit lines	596	1 049	0.039 (0.035)	0.141 (0.081)*
Trade credit	398	1 049	0.103 (0.041)* *	0.202 (0.101)* *
Equity	127	1 049	0.177 (0.062)* **	0.625 (0.140)* **
Grants	427	1 049	0.083 (0.041)* *	0.185 (0.107)*
Leasing	1 143	1 049	0.061 (0.034)*	0.179 (0.081)* *
Factoring	292	1 049	0.078 (0.047)*	0.277 (0.105)* **

NB: The table reports the ATET. The Abadie and Imbens (2016) robust standard error is included in parentheses. Significance levels: * ** *p* < 0.01; * * *p* < 0.05; * *p* < 0.1. Firms can use each source of financing on its own or with others. The control group corresponds to firms that did not obtain or use new financing in the previous year.

*Source:* Authors’ calculations based on PSM results.

Overall, Table 5 shows a positive and significant effect of external sources of financing on innovation. However, not all external sources of financing seem to have the same impact on innovation behaviour. To assess the statistical differences between coefficients, we estimated a *Z*-test. The results reported in Table 6 show that grants are significantly different only when compared with equity financing. In turn, equity financing is significantly different when compared with any other financing instrument, using the count data innovation measure. This means that equity has a higher impact on innovation than the other financing sources and that grants do not have a different effect from any other financing instrument (excluding equity).

**Table 6:** Results Z-test differences between coefficients (ATET): financing in t – 1 in innovation in period t.

H0: differences between coefficients = 0		Being an innovative firm (yes/no)		Number of innovation types (0–4)	
$\beta_{1}$	$\beta_{2}$	|*Z*|	*p*-value	|*Z*|	*p*-value
Grants	Internal funds	0.638	0.523	0.928	0.354
Grants	Bank loans	0.901	0.368	0.122	0.903
Grants	Credit lines	0.816	0.415	0.328	0.743
Grants	Trade credit	0.345	0.730	0.116	0.908
Grants	Equity	1.265	0.206	2.497	0.013 * *
Grants	Leasing	0.413	0.680	0.045	0.964
Grants	Factoring	0.080	0.936	0.614	0.540
Equity	Internal funds	1.810	0.071 *	3.446	0.001 * **
Equity	Bank loans	1.980	0.048 * *	2.653	0.008 * **
Equity	Credit lines	1.938	0.053 *	2.992	0.003 * **
Equity	Trade credit	0.996	0.320	2.450	0.014 * *
Equity	Leasing	1.640	0.101	2.757	0.006 * **
Equity	Factoring	1.272	0.203	1.989	0.047 * *

NB: The *Z*-test was estimated based on Clogg et al. (1995), where ‘H0: differences between coefficients = 0′ and the *Z*-test is estimated as$\;\frac{\beta_{1}-\beta_{2}}{\sqrt{{\left(\mathrm{Standard error}\beta_{1}\right)}^{2}+{\left(\mathrm{Standard error}\beta_{2}\right)}^{2}}}$. Significance levels: * ** *p* < 0.01; * * *p* < 0.05; * *p* < 0.1.

*Source:* Authors’ calculations based on PSM results.

Furthermore, the results regarding equity financing (Table 6) do not confirm the hypothesis that the effect disappears in the short term, 1 year after VC investment, as shown by Engel and Keilbach (2007) and Guo and Jiang (2013). Rather, the present study’s results indicate that the effect is positive and significant 1 year after equity investment. One possible explanation for this divergence could lie in the use of different innovation measures and comparison groups. In the cited studies (Engel and Keilbach, 2007, Guo and Jiang, 2013), the authors used patent applications or R&D investment as the innovation measure, whereas the present study used the OECD and Eurostat (2018) concept of innovation (product, process, organisation and marketing), which usually takes place after patenting and R&D.

As a complementary analysis, we also assessed the effect of financing obtained between April and September 2014 (period *t – 1*) on innovation introduced between October 2013 and September 2014 (period *t – 1*). However, the main limitation of this analysis is that innovation could take place before financing because the questions about financing in SAFE are about the firm’s situation in the last 6 months and innovation behaviour in the previous 12 months. Nevertheless, since there are 6 months where financing could anticipate innovation and because, in some cases, an innovation could be introduced at the same time as finance is accessed (e.g. with internal funds and credit lines), we think that this analysis could complement the results obtained previously. Table 7 reports the effect of different sources of financing on firm innovation behaviour, measured in the same period (*t – 1*).

**Table 7:** Effect of financing in period t – 1 on innovation behaviour in period t – 1.

Source of financing	Number of firms		Being an innovative firm	Number of innovation types
	Treated	Control	yes/no	0–4
Internal funds	663	1 049	0.117 (0.037)* **	0.204 (0.078)* **
External sources	2 599	1 049	0.097 (0.025)* **	0.219 (0.055)* **
Bank loans	596	1 049	0.127 (0.040)* **	0.269 (0.099)* **
Credit lines	596	1 049	0.115 (0.033)* **	0.203 (0.086)* *
Trade credit	398	1 049	0.122 (0.048)* *	0.283 (0.112)* *
Equity	127	1 049	0.210 (0.062)* **	0.537 (0.132)* **
Grants	427	1 049	0.157 (0.044)* **	0.301 (0.110)* **
Leasing	1 143	1 049	0.108 (0.033)* **	0.240 (0.070)* **
Factoring	292	1 049	0.090 (0.056)	0.279 (0.112)* *

NB: The table reports the ATET. The Abadie and Imbens (2016) robust standard error is included in parentheses. Significance levels: * ** *p* < 0.01; * * *p* < 0.05; * *p* < 0.1. Firms can use each source of financing on its own or with others. The control group corresponds to firms that did not obtain or use new financing in the previous year.

*Source:* Authors’ calculations based on PSM results.

The results show that all sources of finance (except factoring with the innovation dummy variable) have a positive effect on the two innovation measures, including internal funds, credit lines and bank loans, about which some doubt existed in the previous analysis. The results in Table 7, compared with those in Table 5, suggest a more significant relationship between financing and innovation in the same period (*t – 1*) than for financing in period *t – 1* and innovation in period *t*, even if the sizes of the coefficients are not statistically different (Table G2 in the supplementary material). However, as we said previously, given the possibility of innovation happening before financing, we choose to focus the output additionality on firm growth based on only the results obtained for period *t*. Nevertheless, the findings reported in Table 7, when compared with Table 5, are interesting. For instance, internal funds seem to be more important for the immediate launch of an innovation in the market (same period), whereas other sources of funding may be more relevant to launching an innovation that requires more planning and more time to be introduced on the market.

### 4.3 Assessing output additionality on firm growth

Regarding output additionality on firm growth as the result of financing, Table 8 summarises the marginal effect of innovation financing on the probability of increasing turnover or the number of employees. On average, using an external source of financing has an additional impact on firm growth; however, as in the previous analysis, not all external sources of financing have an effect or the same effect on increased turnover and employment.

**Table 8:** Complementary log-log regression results: average marginal effect of output additionality on firm growth by source of financing.

	Number of firms	Increase in turnover	Increase in employment
ATE = being an innovative firm (yes/no)			
External financing	2 599	0.054 (0.014)* **	0.035 (0.013)* **
Trade credit	398	0.035 (0.036)	0.030 (0.034)
Equity	127	0.156 (0.061)* **	– 0.055 (0.054)
Grants	427	0.051 (0.032)	0.082 (0.032)* *
Leasing	1 143	0.037 (0.021)*	0.039 (0.019)* *
Factoring	292	0.044 (0.041)	– 0.015 (0.038)
ATE = number of innovations			
External financing	2 599	0.023 (0.006)* **	0.025 (0.005)* **
Bank loans	596	0.024 (0.012)* *	0.033 (0.011)* **
Trade credit	398	0.020 (0.015)	0.013 (0.015)
Credit lines	596	0.029 (0.012)* *	0.032 (0.010)* **
Equity	127	0.054 (0.025)* *	– 0.022 (0.023)
Grants	427	0.031 (0.013)* *	0.029 (0.012)* *
Leasing	1 143	0.021 (0.009)* *	0.023 (0.009)* **
Factoring	292	0.027 (0.017)	0.023 (0.017)

NB: Regressions included firms’ characteristics (size and age and ownership) in period *t*, sector and country/region fixed effect, and growth indicators in period *t – 1*. Only those using the source of finance assessed are included in each regression. Only sources of finance for which ATET is shown to be significant have been assessed. Robust standard errors in parentheses. Significance levels: * ** *p* < 0.01, * * *p* < 0.05 and * *p* < 0.1.

*Source:* Authors’ calculations based on Clog-log results reported in Section E of the supplementary material.

The output additionality on turnover growth is found to be the highest in firms that have issued equity financing and the lowest when linked with leasing. These conclusions are observed for both innovation measures. Grants, credit lines and bank loans also showed significant output additionality on turnover growth, but only when the number of types of innovation is used as an output indicator. These effects are more modest in size than the equity financing effect.

As for output additionality on employment growth, grants and leasing are the only financing instruments with a significant positive effect for the two innovation measures. In both cases, the effect size is more important when firms use grants, but only when compared with leasing. Grants’ robust and positive effect makes sense because the probability of receiving them is generally associated with the likelihood of job creation (Santos et al., 2019), creating an incentive for employment growth. In addition, when leasing is associated with an investment in fixed assets, the additional equipment required for innovation purposes can lead to increased production, which could explain the positive effect of output additionality on employment growth and turnover growth (although of a smaller relative size). Furthermore, bank loans and credit lines also showed significant output additionality on employment growth, but, as with turnover growth, only when the number of innovation types is used. Regarding the size of their effects, they are slightly more significant than for grants.

Finally, equity financing seems to have no additional effect on employment. One possible explanation for this finding could be related to the primary goal of equity investors: maximisation of financial returns. One way to achieve this could be through improving the efficiency of the existing labour force without hiring new employees. Indeed, as Paglia and Harjoto (2014) mention, the impact of equity is not long-lasting and tends to disappear 1 year after investment. Together, these justifications could explain why equity financing shows no additional effect on employment growth.

### 4.4 Complementarity analysis

As a complementary analysis and because firms can use each source of finance on its own or with others (Table 3), the effect of using one or more sources of financing was assessed. Unfortunately, the high number of combinations and the limited observations of each source do not allow a disaggregated analysis. Thus, a variable indicating to what extent each firm used a source of finance was created. Table 9 shows the results of the PSM for different numbers of combinations^16^.

**Table 9:** Effect of different financing in period t – 1 on innovation behaviour in period t.

Number of source of financing	Number of firms		Being an innovative firm	Number of innovations introduced
	Treated	Control	Yes/no	0–4
1	978	1 049	– 0.011 (0.028)	– 0.001 (0.068)
2	788	1 049	0.073 (0.033)* *	0.153 (0.080)*
3	492	1 049	0.127 (0.037)* **	0.292 (0.092)* **
4	291	1 049	0.118 (0.048)* *	0.184 (0.102)*
≥ 5	188	1 049	0.054 (0.063)	0.283 (0.140)* *

NB: The table reports the ATET. The Abadie and Imbens (2016) robust standard error is included in parentheses. Significance levels: * ** *p* < 0.01; * * *p* < 0.05; * *p* < 0.1. The control group corresponds to firms that did not obtain or use new financing in the previous year.

*Source:* Authors’ calculations based on PSM results.

As seen in Table 9, no significant difference between using one source and no source of new financing emerges, whereas using more than one source of financing has a positive and significant impact on innovation. Furthermore, the results of the *Z*-test regarding differences between coefficients (available upon request) are not statistically significant. These conclusions may suggest that financing instruments are complementary to one another rather than substitutes.

Finally, we also estimated the effect of different combinations of financing instruments; the results of the PSM are reported in Table 10.

**Table 10:** Effect of different financing combinations in period t – 1 on innovation behaviour in period t.

Source of financing	Number of firms		Being an innovative firm	Number of innovation types
	Treated	Control	Yes/no	0–4
Bank loans and grants	822	1 049	0.044 (0.032)	0.159 (0.081)* *
Bank loans and equity	702	1 049	0.068 (0.035)*	0.262 (0.080)* **
Internal funds and grants	984	1 049	0.058 (0.035)*	0.137 (0.087)
Internal funds and equity	755	1 049	0.120 (0.035)* **	0.270 (0.080)* **
Internal funds and leasing	1 541	1 049	0.048 (0.025)*	0.050 (0.069)
Leasing and grants	1 408	1 049	0.058 (0.031)*	0.153 (0.078)*
Leasing and equity	1 221	1 049	0.077 (0.028)* **	0.177 (0.080)* *
Credit lines and grants	899	1 049	0.063 (0.032)* *	0.168 (0.075)* *
Credit lines and equity	701	1 049	0.138 (0.033)* **	0.345 (0.075)* **

NB: The table reports the ATET. The Abadie and Imbens (2016) robust standard error is included in parentheses. Significance levels: * ** *p* < 0.01; * * *p* < 0.05; * *p* < 0.1. Firms can use each source of financing on its own or with others. The control group corresponds to firms that did not obtain or use new financing in the previous year.

*Source:* Authors’ calculations based on PSM results.

Regarding the comparison between the sizes of the effects, the results of the *Z*-test (Table G1 in the supplementary material) show that credit lines and equity, which registered the highest ATETs, are statistically significantly different’ from any other combination of grants and any additional financing instrument. Almost all combinations reveal a positive effect on innovation. However, when we assess differences between credit lines along with equity and equity on its own or with another financing instrument, using the count data innovation measure, we see that the ATET of equity financing used on its own or with any other financing instrument is higher than the ATET of credit lines and equity. This can lead to the conclusion that the perfect combination does not exist.

## 5 Conclusions and policy recommendations

The main originality and contribution of the present paper, which is based on a novel database, is the assessment of the effectiveness of eight sources of financing for firms’ innovative activities and, subsequently, the impacts of these activities on output growth in the same period, making their impacts comparable. Previous studies in the literature concentrate more on assessing the effect of only one source of financing (e.g. public support or VC) on innovation or firm growth. However, there are few comparisons of the effects of different sources of finance.

The paper assessed the financing–innovation–growth linkage using a three-step approach. The first two steps were based on the PSM, aiming to evaluate the effect of eight sources of financing on innovation. The last step involved a regression estimation, using a Clog-log model, aiming to quantify the impact of innovation financing, estimated in the first steps, on the probability of firm growth.

The findings presented here support the results of previous studies, demonstrate the importance of external financing for promoting innovation and emphasise that some external financing sources used in one year seem to be more effective than others in stimulating innovation in the subsequent year. Equity financing has a more significant effect on the strategic decision to innovate and the highest output additionality on firm turnover growth compared with the effects of other sources of financing. Grants registered a moderate effect on innovation and output additionality on firm growth (both turnover and employment). Furthermore, grants appear to increase employment more than turnover. Nevertheless, the number of financing instruments used together also seems to matter, and the present study reveals that a financing instrument used alone does not affect innovation.

These new results address a gap in the literature and contribute to a better understanding of which external funding sources, depending on the nature and importance of financing constraints, are the most effective in spurring innovation and, subsequently, economic growth. These findings should be of primary interest to innovative firms in relation to their strategies for financing their innovative activities.

In the pursuit of fostering a robust innovation–growth linkage within the EU’s SMEs, it is imperative to consider a multifaceted approach to financing. The empirical evidence presented in this study underscores the differential impact that various financing sources exert on innovation and subsequent firm growth. This necessitates a nuanced policy framework that recognises the heterogeneity of financing needs across different stages of a firm’s development. First, the policy architecture must advocate the diversification of financing instruments. The study’s insights reveal that equity financing is particularly effective in augmenting turnover growth, suggesting that policies should be conducive to the proliferation of equity markets. This could be operationalised through fiscal incentives aimed at angel investors and venture capitalists, alongside bolstering crowdfunding mechanisms to democratise access to equity for a wider array of enterprises. Second, the facilitation of asset-based financing avenues, such as leasing and factoring, emerges as a critical policy recommendation. Given their utility in addressing the constraints faced by firms that lack tangible collateral, regulatory frameworks should be streamlined to enhance the security and accessibility of these instruments. These measures would empower innovative firms to better manage their working capital, thereby enabling more robust investment in R&D activities. Furthermore, the asymmetry of information between financial entities and innovative firms presents a significant barrier to accessing finance. Policies aimed at enhancing information symmetry could include the establishment of comprehensive credit-reporting systems and the implementation of rigorous due diligence processes. These initiatives would serve to mitigate the information gap, thereby facilitating more equitable access to finance.

Government interventions in the form of grants and subsidies also warrant a strategic approach. The potential for this support to inadvertently crowd out private R&D investment necessitates a complementary design, targeting high-risk innovation projects that may not attract private funding. This tailored support should aim to catalyse innovation without displacing private-sector investment.

The evidence of complementarities among different financing sources suggests that policies promoting their combined utilisation could amplify their collective impact on innovation and growth. This policy stance would encourage firms to leverage the synergistic potential of diverse financing instruments, optimising their overall growth trajectory. Moreover, the dynamic nature of finance and innovation interactions calls for ongoing monitoring and evaluation. Policymakers should commit to a continuous assessment of the effectiveness of financial instruments, adapting and evolving the financial ecosystem in response to empirical findings. Last, the complexity inherent in the financing landscape necessitates a commitment to enhancing financial literacy among SMEs. Policy initiatives should support educational programmes and advisory services that equip firms with the knowledge and skills to navigate and exploit the array of available financing options effectively.

In conclusion, the policy recommendations derived from this study advocate a comprehensive and adaptive approach to financing innovation in SMEs. This approach should be characterised by diversity, specificity and responsiveness, ensuring that the financing–innovation–growth nexus is optimally supported for the sustained economic advancement of the EU.

Further research should focus on understanding why subsidies are less effective, namely analysing the effectiveness of the selection procedure for public support and evaluating subsidised firms’ fulfilment of the financial objectives set out in the application forms.

## Disclaimer

The views expressed are purely those of the authors and may not in any circumstances be regarded as stating an official position of the European Commission.

## Data statement

The research data is confidential.

## Footnotes

1 In the literature, the terms ‘impact’ and ‘causal effect’ are generally used interchangeably. Nevertheless, taking a cautious attitude, we prefer to speak in this paper not of ‘direct casual effect’, but of the ‘impact’ of financing sources on innovation and growth. Impact can possibly also include (hopefully, small) ‘indirect’ effects induced by non-controlled confounders, although we believe that the extent of these indirect effects is minimal when a proper set of observable confounders is taken into account as we did in our evaluation setting.

2 Except Yano and Shiraishi (2020), who analyse the effects of internal finance and external finance on intangible assets of Chinese firms.

3 This asymmetric information problem refers to the information gap between entrepreneurs/inventors and potential investors about the innovative content of an R & D project (Hall, 2009, OECD, 2012, OECD, 2015a).

4 ‘Lessor’ refers to the entity that leases the equipment or property to the ‘lessee’, who, in turn, makes use of it.

5 For example, if a firm needs a new car, it can choose to pay the whole amount when it does the deal or to pay in 48 monthly instalments though a leasing arrangement.

6 Crowding out happens when all or part of the subsidy is used to replace firms’ own R&D expenditure and, in the end, they invest less than they would have without the subsidy (Dimos and Pugh, 2016).

7 No impact of public subsidy means that subsidised firms and non-subsidised ones have the same net innovation performance.

8 Since the survey is anonymous, cross-referencing with other databases is not possible.

9 When we started the study, these were the last waves available. We chose to work with data from only the first round of each year because the second rounds have a more limited geographic coverage (euro area only) and do not include questions related to the past performance of firms and innovative behaviour.

10 Increasing the number of waves significantly reduces the number of complete observations; for this reason, we decided to use only two waves.

11 It is also important to note that exploiting a time lag from treatment to outcome allows us to attenuate the simultaneous bias between treatment and outcome. Moreover, this makes our analysis closer to a difference-in-differences model. However, it goes without saying that we cannot test the presence of a pre-treatment common trend. The use of a large set of observable covariates and the obtaining of a good balance using our matching procedure are, however, good signals that the ceteris paribus condition implied by the (untestable, in our case) common trend is likely to be met.

12 Results of the collinearity diagnostic are reported in Table C1 in the supplementary material.

13 The balancing quality could be tested through visual analysis of kernel density plots of treated and control groups, allowing us to see bias correction before and after matching. A covariate is balanced when its distribution does not vary between groups after matching.

14 In fact, the conditional ATET is a firm idiosyncratic variable, that is, each firm has a specific ATET that is conditional on its characteristics (i.e. the covariates).

15 Region refers to a group of geographically close countries. Given the limited number of observations for each of the EU-28 after selecting only treated firms, it is not possible to implement a country fixed effect for all of them.

16 See the balancing test in Figure F1 (supplementary material).
