# Supplementary material for: Sources of financing: Which ones are more effective in innovation–growth linkage?
Source: Econ Syst. 2024 Jun;48(2):None. doi: 10.1016/j.ecosys.2023.101177 (PMC11188937; doi:10.1016/j.ecosys.2023.101177)
Supplement: Supplementary file 1 — Supplementary material. [file mmc1.pdf]

# Supplement material

## A. Variable description

Table A1. Variable name and description

| Variable name | Variable description |
|---|---|
| **Source of financing (INPUT)** | |
| *Financing: internal funds* | = 1 if firm has retained earnings or sold assets in the past 6 months (question 4A in SAFE questionnaire); 0 otherwise. |
| *Financing: external source* | = 1 if firm has obtained or used any source of external financing in the past 6 months (question 4A and 7B in SAFE questionnaire); 0 otherwise.<br>External source of financing refers to bank loan, trade credit, credit line, grants, equity, leasing, factoring, debt securities and all others not individually identified (loans from family, friends, related enterprise or shareholders, subordinated debt instruments, participating loans, peer-to-peer lending and crowdfunding). |
| *No-financing* | = if firm has not used, issued or obtained any sources of external financing, among those listed above, in the past 6 months (question 4A and 7B in SAFE questionnaire); 0 otherwise (= control group and untreated). |
| *Financing: bank loan* | = 1 if firm has obtained finance through a bank loan over the past 6 months; 0 otherwise. Obtained means to apply for finance and received everything requested, most of it or a limited part (question 7B in SAFE questionnaire). |
| *Financing: trade credit* | = 1 if firm has obtained finance through a trade credit over the past 6 months; 0 otherwise. Obtained means to apply for finance and received everything requested, most of it or a limited part (question 7B in SAFE questionnaire). |
| *Financing: credit line* | = 1 if firm has obtained finance through a credit line, bank overdraft or credit card overdraft over the past 6 months; 0 otherwise. Obtained means to apply for finance and received everything requested, most of it or a limited part (question 7B in SAFE questionnaire). |
| *Financing: grants* | = 1 if firm has obtained new grants or subsidized bank loan in the past 6 months (question 4A in SAFE questionnaire); 0 otherwise. |
| *Financing: equity* | = 1 if firm has issued equity capital, including venture capital and business angels, in the past 6 months (question 4A in SAFE questionnaire); 0 otherwise. |
| *Financing: leasing* | = 1 if firm has used leasing or hire-purchase in the past 6 months (question 4A in SAFE questionnaire); 0 otherwise. |
| *Financing: factoring* | = if firm has issued factoring in the past 6 months (question 4A in SAFE questionnaire); 0 otherwise. |
| *N° of financing* | N° of external financing sources used by firms (0-7), among those listed above, in the past 6 months. Zero means no financing used or obtained. |
| **Innovation indicator (OUTPUT)** | |
| *Innovative firm* | = 1 if firm has introduced, during the past 12 months, one of the following types of innovative activities: a) new or improved product or service; b) new or improved production process or method; c) new organization of management; d) new way of selling goods or services; 0 otherwise. |
| *N° of innovations* | N° of innovation types introduced by firms (0-4), among those listed above, during the past 12 months. Zero means no innovation has been introduced in the market and firm is considered non-innovative. |
| **Firm growth indicators (OUTCOME)** | |
| Increase: turnover | = 1 if firm has increased its turnover in the past 6 months; 0 otherwise (= remained unchanged or decreased). |
| Increase: employees | = 1 if firm has increased the number of employees in the past 6 months; 0 otherwise (= remained unchanged or decreased). |

Continued on the next page…

Table A1. Variable name and description (continuation)

| Variable name | Variable description |
|---|---|
| **EXPLANATORY VARIABLES OR COVARIANTS** | |
| **Firm size** | |
| Firm size was divided in three categories (micro, small and medium) taking turnover into account, as reported in the **Commission Recommendation 2003/361.** | |
| *Size: Micro* | = 1 if micro firm; 0 otherwise (reference category). |
| *Size: Small* | = 1 if small firm; 0 otherwise. |
| *Size: Medium* | = 1 if medium-sized firm; 0 otherwise. |
| **Firm's main activity sector** | |
| *Activity: Industry* | = 1 if firm's main activity is industry, which includes manufacturing, mining and electricity, gas and water supply; 0 otherwise. |
| *Activity: Trade* | = 1 if firm's main activity is wholesale or retail trade; 0 otherwise. |
| *Activity: Services* | = 1 if firm's main activity is services to businesses or individuals, for example hotels and restaurants, IT services; 0 otherwise (reference category). |
| *Activity: Construction* | = 1 if firm's main activity is construction; 0 otherwise. |
| **Firm's ownership** | |
| *Ownership: private* | = 1 if firm's main ownership lies with one owner only, family or entrepreneurs; 0 otherwise (reference category). |
| *Ownership: public shareholders* | = 1 if firm's main ownership lies with public shareholders, as firm is listed on the stock market; 0 otherwise. |
| *Ownership: other* | = 1 if firm's main ownership lies with other enterprises; venture capital enterprises or business angels; with other business associates or others not previously listed; 0 otherwise (reference category). |
| *Autonomy* | = 1 if firm is an autonomous profit-oriented enterprise, making independent financial decisions; 0 otherwise (= a subsidiary or a branch of another enterprise). |
| **Firm age** | |
| Firm age was divided in three categories taking into account the criteria of **Criscuolo *et al.*** (2014). | |
| *Age: Young firm* | = 1 if under 5 years old; 0 otherwise (reference category). |
| *Age: Mature firm* | = 1 if a mature firm between 5 and 10 years old; 0 otherwise (reference category). |
| *Age: Old firm* | = 1 if an old firm more than 10 years old; 0 otherwise. |
| **Firm's past performance** | |
| *Export intensity* | % of turnover destined to international market or non-residents in the year before the survey (e.g. refers to value of 2013 for survey made in 2014). |
| *Grow fast* | = 1 if firm has grown in terms of turnover, on average over 20% per year, in the past three years (2011 – 2013); 0 otherwise (reference category). |
| *Grow moderate* | = 1 if firm has grown in terms of turnover less than 20% per year, in the past three years (2011 – 2013) ; 0 otherwise. |
| *No growth* | = 1 if firm has not grown or become smaller in terms of turnover, in the past three years (2011 – 2013); 0 otherwise. |

Source: Authors' own elaboration based on SAFE Survey template.

## B. Descriptive Statistic

Table B1. Mean, Standard Deviation, Minimum and Maximum all sample

| Variables | Mean | Std. Dev. | Min | Max |
|---|---|---|---|---|
| Size: Micro | 0.5378 | 0.4986 | 0 | 1 |
| Size: Small | 0.2755 | 0.4468 | 0 | 1 |
| Size: Medium | 0.1867 | 0.3898 | 0 | 1 |
| Age: Young firm | 0.0742 | 0.2622 | 0 | 1 |
| Age: Mature firm | 0.1302 | 0.3366 | 0 | 1 |
| Age: Old firm | 0.7956 | 0.4033 | 0 | 1 |
| Ownership: Autonomy | 0.8936 | 0.3084 | 0 | 1 |
| Ownership: Private | 0.8365 | 0.3699 | 0 | 1 |
| Ownership: Public shareholders | 0.0217 | 0.1456 | 0 | 1 |
| Ownership: Other | 0.1418 | 0.3489 | 0 | 1 |
| Past performance: Export intensity (%) | 0.1811 | 0.2931 | 0 | 1 |
| Past performance: Grow fast | 0.1492 | 0.3564 | 0 | 1 |
| Past performance: Grow moderate | 0.4099 | 0.4919 | 0 | 1 |
| Past performance: No growth | 0.4408 | 0.4966 | 0 | 1 |
| Output: Being an innovative firm | 0.5964 | 0.4907 | 0 | 1 |
| Output: N° of different innovations | 1.0800 | 1.2201 | 0 | 4 |
| Outcome: Increasing turnover | 0.4229 | 0.4941 | 0 | 1 |
| Outcome: Increasing employment | 0.2520 | 0.4342 | 0 | 1 |
| Activity: Industry | 0.2763 | 0.4472 | 0 | 1 |
| Activity: Construction | 0.1149 | 0.3189 | 0 | 1 |
| Activity: Trade | 0.2845 | 0.4512 | 0 | 1 |
| Activity: Services | 0.3244 | 0.4682 | 0 | 1 |
| Country: Austria | 0.0304 | 0.1716 | 0 | 1 |
| Country: Belgium | 0.0277 | 0.1642 | 0 | 1 |
| Country: Bulgaria | 0.0306 | 0.1724 | 0 | 1 |
| Country: Cyprus | 0.0048 | 0.0688 | 0 | 1 |
| Country: Czech Republic | 0.0248 | 0.1556 | 0 | 1 |
| Country: Germany | 0.0724 | 0.2591 | 0 | 1 |
| Country: Denmark | 0.0225 | 0.1482 | 0 | 1 |
| Country: Estonia | 0.0034 | 0.0585 | 0 | 1 |
| Country: Spain | 0.0927 | 0.2901 | 0 | 1 |
| Country: Finland | 0.0357 | 0.1855 | 0 | 1 |
| Country: France | 0.1054 | 0.3071 | 0 | 1 |
| Country: Greece | 0.0386 | 0.1926 | 0 | 1 |
| Country: Croatia | 0.0116 | 0.1072 | 0 | 1 |
| Country: Hungary | 0.0275 | 0.1635 | 0 | 1 |
| Country: Ireland | 0.0283 | 0.1657 | 0 | 1 |
| Country: Italy | 0.1305 | 0.3369 | 0 | 1 |
| Country: Lithuania | 0.0114 | 0.1060 | 0 | 1 |
| Country: Luxembourg | 0.0055 | 0.0743 | 0 | 1 |
| Country: Latvia | 0.0079 | 0.0887 | 0 | 1 |
| Country: Malta | 0.0055 | 0.0743 | 0 | 1 |
| Country: Netherlands | 0.0523 | 0.2227 | 0 | 1 |
| Country: Poland | 0.0663 | 0.2488 | 0 | 1 |
| Country: Portugal | 0.0320 | 0.1759 | 0 | 1 |
| Country: Romania | 0.0225 | 0.1482 | 0 | 1 |
| Country: Sweden | 0.0225 | 0.1482 | 0 | 1 |
| Country: Slovenia | 0.0082 | 0.0901 | 0 | 1 |
| Country: Slovakia | 0.0235 | 0.1515 | 0 | 1 |
| Country: United Kingdom | 0.0557 | 0.2294 | 0 | 1 |

Source: Authors' own elaboration.
Note: Number of observations = 3,786.

Table B2. Source of financing, mean by country

| Country | Internal | Grants | Equity | Leasing | Factoring | Bank loan | Trade credit | Credit line |
|---|---|---|---|---|---|---|---|---|
| EU28 | 0.18 | 0.11 | 0.03 | 0.30 | 0.08 | 0.16 | 0.11 | 0.16 |
| AT | 0.18 | 0.08 | 0.02 | 0.37 | 0.03 | 0.22 | 0.04 | 0.18 |
| BE | 0.14 | 0.10 | 0.04 | 0.24 | 0.07 | 0.25 | 0.08 | 0.14 |
| BG | 0.19 | 0.11 | 0.06 | 0.25 | 0.02 | 0.13 | 0.07 | 0.16 |
| CY | 0.17 | 0.17 | 0.00 | 0.11 | 0.11 | 0.00 | 0.06 | 0.00 |
| CZ | 0.23 | 0.20 | 0.00 | 0.29 | 0.12 | 0.12 | 0.03 | 0.19 |
| DE | 0.19 | 0.14 | 0.02 | 0.49 | 0.04 | 0.15 | 0.02 | 0.09 |
| DK | 0.08 | 0.05 | 0.12 | 0.39 | 0.04 | 0.06 | 0.01 | 0.13 |
| EE | 0.31 | 0.08 | 0.00 | 0.31 | 0.15 | 0.00 | 0.08 | 0.08 |
| EL | 0.03 | 0.09 | 0.07 | 0.12 | 0.05 | 0.10 | 0.06 | 0.05 |
| ES | 0.14 | 0.15 | 0.02 | 0.20 | 0.11 | 0.24 | 0.29 | 0.22 |
| FI | 0.19 | 0.04 | 0.02 | 0.42 | 0.10 | 0.19 | 0.10 | 0.07 |
| FR | 0.24 | 0.08 | 0.03 | 0.34 | 0.14 | 0.27 | 0.04 | 0.21 |
| HR | 0.23 | 0.16 | 0.07 | 0.39 | 0.09 | 0.25 | 0.00 | 0.16 |
| HU | 0.12 | 0.10 | 0.01 | 0.23 | 0.02 | 0.07 | 0.02 | 0.16 |
| IE | 0.29 | 0.14 | 0.04 | 0.30 | 0.12 | 0.06 | 0.24 | 0.13 |
| IT | 0.20 | 0.18 | 0.01 | 0.14 | 0.06 | 0.21 | 0.11 | 0.21 |
| LT | 0.21 | 0.07 | 0.09 | 0.33 | 0.09 | 0.07 | 0.02 | 0.02 |
| LU | 0.14 | 0.00 | 0.10 | 0.19 | 0.00 | 0.10 | 0.10 | 0.29 |
| LV | 0.03 | 0.07 | 0.10 | 0.43 | 0.00 | 0.07 | 0.00 | 0.10 |
| MT | 0.33 | 0.05 | 0.05 | 0.33 | 0.10 | 0.14 | 0.10 | 0.14 |
| NL | 0.11 | 0.02 | 0.02 | 0.38 | 0.06 | 0.04 | 0.05 | 0.04 |
| PL | 0.11 | 0.15 | 0.01 | 0.37 | 0.09 | 0.13 | 0.19 | 0.23 |
| PT | 0.04 | 0.14 | 0.00 | 0.19 | 0.06 | 0.17 | 0.17 | 0.19 |
| RO | 0.16 | 0.06 | 0.01 | 0.25 | 0.06 | 0.12 | 0.14 | 0.18 |
| SE | 0.29 | 0.11 | 0.07 | 0.51 | 0.12 | 0.08 | 0.02 | 0.05 |
| SI | 0.13 | 0.13 | 0.03 | 0.29 | 0.00 | 0.13 | 0.00 | 0.16 |
| SK | 0.17 | 0.02 | 0.27 | 0.31 | 0.04 | 0.06 | 0.01 | 0.19 |
| UK | 0.27 | 0.09 | 0.03 | 0.45 | 0.10 | 0.07 | 0.20 | 0.12 |

Source: Authors' own elaboration.
Note: Number of observations = 3,786.

Table B3. Output: mean by country

| Country | Innovation (Y/N) | Increase turnover (Y/N) | Increase employement (Y/N) |
|---|---|---|---|
| EU28 | 0.60 | 0.42 | 0.25 |
| AT | 0.55 | 0.37 | 0.22 |
| BE | 0.50 | 0.37 | 0.20 |
| BG | 0.58 | 0.34 | 0.22 |
| CY | 0.83 | 0.28 | 0.22 |
| CZ | 0.72 | 0.52 | 0.30 |
| DE | 0.50 | 0.49 | 0.25 |
| DK | 0.60 | 0.55 | 0.38 |
| EE | 0.54 | 0.15 | 0.15 |
| EL | 0.64 | 0.24 | 0.18 |
| ES | 0.62 | 0.51 | 0.32 |
| FI | 0.78 | 0.33 | 0.24 |
| FR | 0.60 | 0.34 | 0.21 |
| HR | 0.64 | 0.39 | 0.18 |
| HU | 0.35 | 0.48 | 0.30 |
| IE | 0.68 | 0.62 | 0.33 |
| IT | 0.60 | 0.34 | 0.19 |
| LT | 0.53 | 0.47 | 0.26 |
| LU | 0.57 | 0.38 | 0.33 |
| LV | 0.60 | 0.40 | 0.20 |
| MT | 0.67 | 0.48 | 0.52 |
| NL | 0.60 | 0.54 | 0.27 |
| PL | 0.55 | 0.35 | 0.24 |
| PT | 0.64 | 0.41 | 0.26 |
| RO | 0.74 | 0.55 | 0.36 |
| SE | 0.66 | 0.41 | 0.28 |
| SI | 0.74 | 0.48 | 0.26 |
| SK | 0.63 | 0.47 | 0.25 |
| UK | 0.52 | 0.53 | 0.31 |

Source: Authors’ own elaboration.
Note: Number of observations = 3,786.

## C. Testing correlation and multi-collinearity

Table C1. Correlation matrix and collinearity diagnostics (covariants in PS)

| # | Variables | VIF | Correlation matrix | | | | | | | | | | | |
|---|---|---|---|---|---|---|---|---|---|---|---|---|---|---|
| | | | 1 | 2 | 3 | 4 | 5 | 6 | 7 | 8 | 9 | 10 | 11 | 12 |
| 1 | Size: Small | 1.20 | 1.000 | | | | | | | | | | | |
| 2 | Size: Medium | 1.37 | -0.296 | 1.000 | | | | | | | | | | |
| 3 | Age: Old firm | 1.11 | 0.106 | 0.127 | 1.000 | | | | | | | | | |
| 4 | Ownership: Autonomy | 1.29 | -0.031 | -0.263 | -0.011 | 1.000 | | | | | | | | |
| 5 | Ownership: Private | 1.37 | -0.068 | -0.182 | 0.004 | 0.417 | 1.000 | | | | | | | |
| 6 | Ownership: Public shareholders | 1.13 | 0.006 | 0.073 | -0.001 | -0.119 | -0.337 | 1.000 | | | | | | |
| 7 | Performance: Export intensity | 1.19 | 0.063 | 0.200 | -0.007 | -0.172 | -0.118 | 0.059 | 1.000 | | | | | |
| 8 | Performance: Grow moderate | 2.33 | 0.064 | 0.134 | 0.051 | -0.043 | -0.034 | 0.031 | 0.048 | 1.000 | | | | |
| 9 | Performance: No growth | 2.35 | -0.051 | -0.101 | 0.110 | 0.029 | 0.032 | -0.037 | -0.094 | -0.740 | 1.000 | | | |
| 10 | Activity: Industry | 1.52 | 0.107 | 0.198 | 0.107 | -0.118 | -0.083 | 0.026 | 0.324 | 0.020 | -0.006 | 1.000 | | |
| 11 | Activity: Construction | 1.22 | -0.015 | -0.030 | -0.004 | 0.044 | 0.041 | -0.002 | -0.117 | -0.043 | 0.027 | -0.223 | 1.000 | |
| 12 | Activity: Trade | 1.37 | -0.034 | -0.027 | -0.011 | 0.037 | 0.062 | -0.013 | -0.158 | -0.040 | 0.051 | -0.390 | -0.227 | 1.000 |

Source: Authors' own elaboration.

Note: Number of observations = 3,786.

## D. Balancing test – Type of financing

Figure D1. Distribution of treated and non-treated firms according to the covariates by source of financing

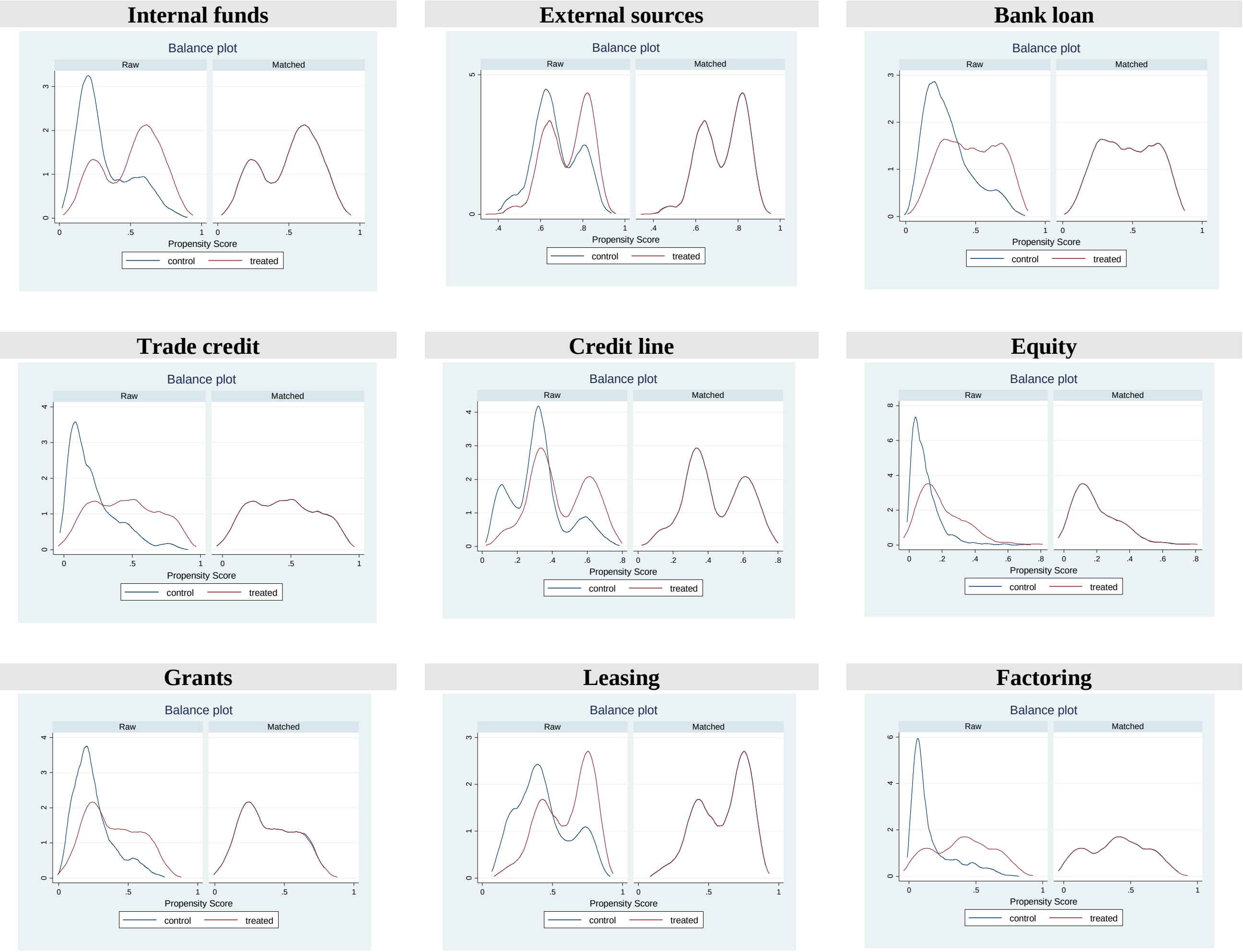


Source: Authors' own elaboration based on results of kernel density plots of treated and control group, before and after matching.

## E. Results of Cloglog regression estimation, by financing source

Table E1. Probability of increasing turnover with dummy innovation measure

| Variables | External financing | Trade credit | Equity | Grants | Leasing | Factoring |
|---|---|---|---|---|---|---|
| | **Model 1** | **Model 2** | **Model 3** | **Model 4** | **Model 5** | **Model 6** |
| *ATE*: Effect of financing on innovation | 0.177 *** | 0.109 | 0.549 ** | 0.172 + | 0.116 * | 0.140 |
| | (0.0466) | (0.113) | (0.232) | (0.111) | (0.0653) | (0.131) |
| Past performance (ΔY), Size and Age | YES | YES | YES | YES | YES | YES |
| Sector and country-region fixed effect | YES | YES | YES | YES | YES | YES |
| Constant | YES | YES | YES | YES | YES | YES |
| N.° observations | 2,599 | 398 | 127 | 427 | 1,143 | 292 |
| Log pseudo-likelihood | -1661.20 | -253.92 | -73.27 | -254.13 | -743.89 | -185.78581 |
| Wald test (H0: All coefficient = 0) | 235.5 *** | 41.72 *** | 25.8 ** | 85.87 *** | 88.25 *** | 32.14 *** |
| Mean VIF | 1.75 | 2.11 | 1.75 | 1.85 | 1.8 | 2.43 |
| Wald test (H0: model has no omitted variables) - chi2(3) | 4.19 | 2.35 | 2.82 | 3.54 | 4.34 | 5.00 |

Source: Authors' own elaboration.
Note: Robust standard errors are reported in parenthesis. Significance level: *** p<0.01, ** p<0.05, * p<0.1, + p<0.15

Table E2. Probability of increasing employment with dummy innovation measure

| Variables | External financing | Trade credit | Equity | Grants | Leasing | Factoring |
|---|---|---|---|---|---|---|
| | **Model 7** | **Model 8** | **Model 9** | **Model 10** | **Model 11** | **Model 12** |
| *ATE*: Effect of financing on innovation | 0.162 *** | 0.127 | -0.247 | 0.331 ** | 0.165 ** | -0.0695 |
| | (0.0601) | (0.142) | (0.245) | (0.132) | (0.0793) | (0.180) |
| Past performance (ΔY), Size and Age | YES | YES | YES | YES | YES | YES |
| Sector and country-region fixed effect | YES | YES | YES | YES | YES | YES |
| Constant | YES | YES | YES | YES | YES | YES |
| N.° observations | 2,599 | 398 | 127 | 427 | 1,143 | 292 |
| Log pseudo-likelihood | -1,374.88 | -220.24 | -68.08 | -243.16 | -645.77 | -150.584 |
| Wald test (H0: All coefficient = 0) | 303.58 *** | 71.54 *** | 21.00 + | 62.96 *** | 135.95 *** | 42.57 *** |
| Mean VIF | 1.73 | 2.07 | 1.69 | 1.82 | 1.77 | 2.4 |
| Wald test (H0: model has no omitted variables) - chi2(3) | 4.36 | 7.05 * | 4.41 | 3.21 | 1.5 | 1.64 |

Source: Authors' own elaboration.
Note: Robust standard errors are reported in parenthesis. Significance level: *** p<0.01, ** p<0.05, * p<0.1, + p<0.15

Table E3. Probability of increasing turnover with count data innovation measure

| Variables | External financing | Bank Loan | Trade credit | Credit line | Equity | Grants | Leasing | Factoring |
|---|---|---|---|---|---|---|---|---|
| | **Model 13** | **Model 14** | **Model 15** | **Model 16** | **Model 17** | **Model 18** | **Model 19** | **Model 20** |
| *ATE*: Effect of financing on innovation | 0.0773 *** | 0.0839 ** | 0.0618 | 0.0966 ** | 0.186 ** | 0.106 ** | 0.0674 ** | 0.0879 |
| | (0.0194) | (0.0405) | (0.0473) | (0.0397) | (0.0912) | (0.0457) | (0.0287) | (0.0556) |
| Past performance (ΔY), Size and Age | YES | YES | YES | YES | YES | YES | YES | YES |
| Sector and country-region fixed effect | YES | YES | YES | YES | YES | YES | YES | YES |
| Constant | YES | YES | YES | YES | YES | YES | YES | YES |
| N.° observations | 2599 | 596 | 398 | 596 | 127 | 427 | 1143 | 292 |
| Log pseudo-likelihood | -1660.36 | -359.06 | -253.49 | -378.08 | -74.08 | -252.56 | -742.66 | -185.09 |
| Wald test (H0: All coefficient = 0) | 237.41 *** | 100.53 *** | 42.09 *** | 60.24 *** | 24.5 ** | 86.81 *** | 90.62 *** | 33.64 *** |
| Mean VIF | 1.75 | 1.91 | 2.11 | 1.77 | 1.76 | 1.85 | 1.8 | 2.43 |
| Wald test (H0: model has no omitted variables) - chi2(3) | 3.69 | 0.76 | 2.23 | 0.48 | 5.25 | 2.54 | 5.3 | 5.73 |

Source: Authors' own elaboration.
Note: Robust standard errors are reported in parenthesis. Significance level: *** p<0.01, ** p<0.05, * p<0.1;

Table E4. Probability of increasing employment with count data innovation measure

| Variables | External financing | Bank Loan | Trade credit | Credit line | Equity | Grants | Leasing | Factoring |
|---|---|---|---|---|---|---|---|---|
| | **Model 21** | **Model 22** | **Model 23** | **Model 24** | **Model 25** | **Model 26** | **Model 27** | **Model 28** |
| *ATE*: Effect of financing on innovation | 0.119 *** | 0.145 *** | 0.0544 | 0.158 *** | -0.0993 | 0.116 ** | 0.0948 *** | 0.111 |
| | (0.0249) | (0.0485) | (0.0626) | (0.0508) | (0.107) | (0.0504) | (0.0362) | (0.0797) |
| Past performance (ΔY), Size and Age | YES | YES | YES | YES | YES | YES | YES | YES |
| Sector and country-region fixed effect | YES | YES | YES | YES | YES | YES | YES | YES |
| Constant | YES | YES | YES | YES | YES | YES | YES | YES |
| N.° observations | 2599 | 596 | 398 | 596 | 127 | 427 | 1143 | 292 |
| Log pseudo-likelihood | -1366.72 | -323.47 | -220.22 | -303.85 | -68.11 | -243.75 | -644.22 | -149.59 |
| Wald test (H0: All coefficient = 0) | 317.76 *** | 82.24 *** | 71.82 *** | 89.37 *** | 20.98 + | 66.57 *** | 138.35 *** | 42.57 *** |
| Mean VIF | 1.73 | 1.88 | 2.06 | 1.73 | 1.70 | 1.82 | 1.77 | 2.4 |
| Wald test (H0: model has no omitted variables) - chi2(3) | 1.47 | 7.71 * | 6.43 * | 4.18 | 8.16 ** | 6.72 * | 1.38 | 1.35 |

Source: Authors' own elaboration.
Note: Robust standard errors are reported in parenthesis. Significance level: *** p<0.01, ** p<0.05, * p<0.1, + p<0.15

## F. Balancing test – N.° financing

Figure F1. Distribution of treated and non-treated firms according to the covariates by n° financing instruments used together

**N° financing = 1**

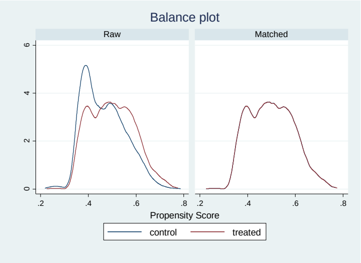


**N° financing = 2**

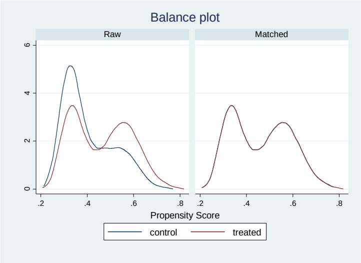


**N° financing = 3**

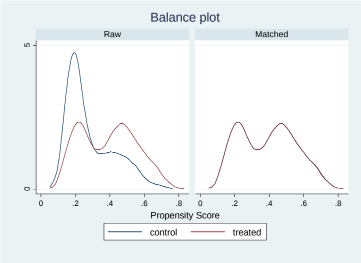


**N° financing = 4**

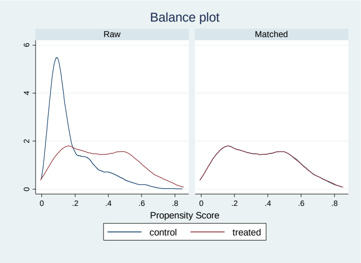


**N° financing ≥ 5**

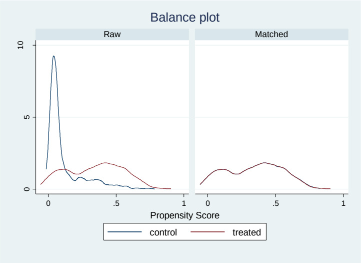


Source: Authors' own elaboration based on results of kernel density plots of treated and control group, before and after matching.

## G. Z-test differences between coefficients

Table G1. Results Z-test differences between coefficients (*ATET*): combination of financing instruments

| *H0: Differences between coefficients = 0* | | Being an innovative Firm (Yes/No) | | | N° of different Innovations type (0 – 4) | | |
|---|---|---|---|---|---|---|---|
| $\boldsymbol{\beta_1}$ | $\beta_2$ | \|Z\| | p-value | | \|Z\| | p-value | |
| Bank loans and grants | Bank loans and equity | 0.495 | 0.6204 | | 0.914 | 0.3610 | |
| Internal funds and grants | Internal funds and equity | 1.253 | 0.2104 | | 1.124 | 0.2611 | |
| Leasing and grants | Leasing and equity | 0.458 | 0.6468 | | 0.222 | 0.8240 | |
| Credit line and grants | Credit line and equity | 1.626 | 0.1042 | + | 1.662 | 0.0968 | * |
| Credit line and equity | Bank loans and grants | 2.036 | 0.0420 | ** | 1.690 | 0.0914 | * |
| Credit line and equity | Internal funds and grants | 1.662 | 0.0968 | * | 1.805 | 0.0713 | * |
| Credit line and equity | Leasing and grants | 1.756 | 0.0793 | * | 1.771 | 0.0768 | * |
| Credit line and equity | Bank loans and equity | 1.465 | 0.1433 | + | 0.757 | 0.4492 | |
| Credit line and equity | Internal funds and equity | 0.376 | 0.7070 | | 0.677 | 0.4987 | |
| Credit line and equity | Leasing and equity | 1.394 | 0.1637 | | 1.524 | 0.1279 | + |
| Bank loans and equity | Internal funds and grants | 0.192 | 0.8476 | | 1.059 | 0.2899 | |
| Bank loans and equity | Leasing and grants | 0.200 | 0.8412 | | 0.981 | 0.3269 | |
| Bank loans and equity | Credit line and grants | 0.097 | 0.9231 | | 0.858 | 0.3912 | |
| Internal funds and grants | Bank loans and grants | 0.296 | 0.7676 | | 0.180 | 0.8568 | |
| Internal funds and grants | Leasing and grants | 0.002 | 0.9980 | | 0.132 | 0.8949 | |
| Internal funds and grants | Credit line and grants | 0.103 | 0.9177 | | 0.270 | 0.7873 | |
| Credit line and grants | Bank loans and grants | 0.416 | 0.6772 | | 0.087 | 0.9304 | |
| Credit line and grants | Leasing and grants | 0.107 | 0.9148 | | 0.144 | 0.8858 | |
| Bank loans and grants | Leasing and grants | 0.316 | 0.7521 | | 0.053 | 0.9578 | |
| Equity | Credit line and equity | 0.555 | 0.5788 | | 1.762 | 0.0783 | * |

Source: Authors' own elaboration based on PSM results.
Note: The Z-test was estimated following **Clogg *et al.*** (1995) approach , where *H0: Differences between coefficients = 0* and the Z test is estimated by the following equation: $Z = \frac{\beta_1 - \beta_2}{\sqrt{(Std.Error\ \beta_1)^2 + (Std.Error\ \beta_2)^2}}$

Table G2. Results of Z-test for testing statistical differences between coefficients in Table 5 and Table 7

| Source of financing | Being an innovative firm in T-1 *versus* in T | | N° of innovations introduced in T-1 *versus* in T | |
|---|---|---|---|---|
| | Z | p-value | Z | p-value |
| Internal funds | 1.353 | 0.176 | 1.258 | 0.209 |
| External sources | 1.270 | 0.204 | 0.540 | 0.589 |
| Bank loan | 1.701 | 0.089 | 0.721 | 0.471 |
| Credit line | 1.580 | 0.114 | 0.525 | 0.600 |
| Trade credit | 0.301 | 0.763 | 0.537 | 0.591 |
| Equity | 0.376 | 0.707 | 0.457 | 0.648 |
| Grants | 1.230 | 0.219 | 0.756 | 0.450 |
| Leasing | 0.992 | 0.321 | 0.570 | 0.569 |
| Factoring | 0.164 | 0.870 | 0.013 | 0.990 |

Source: Authors' own elaboration based on results of Tables 5 and 7.
Note: The Z-test was estimated based on **Clogg *et al.*** (1995), where *H0: Differences between coefficients = 0* and the Z test is estimated by the following equation: $= \frac{\beta_1 - \beta_2}{\sqrt{(Std.Error\ \beta_1)^2 + (Std.Error\ \beta_2)^2}}$ . Significance level: *** p<0.01, ** p<0.05, * p<0.1.
